# Prevalence and distribution of Human Papillomavirus (HPV) genotypes among HIV infected women in Lomé, Togo

**DOI:** 10.1371/journal.pone.0212516

**Published:** 2019-02-27

**Authors:** Yawo Tufa Nyasenu, Fifonsi Adjidossi Gbeasor-Komlanvi, Amivi Ehlan, Sabi Abdul-Raouf Issa, Sika Dossim, Malewe Kolou, Brice Martial Yambiyo, Mireille Prince-David, Mounerou Salou, Didier Koumavi Ekouevi, Anoumou Dagnra

**Affiliations:** 1 Département des Sciences Fondamentales, Université de Lomé, Lomé, Togo; 2 Département de Santé Publique, Université de Lomé, Lomé, Togo; 3 Centre Africain de Recherche en Epidémiologie et en Santé Publique, Lomé, Togo; 4 Service d’Epidémiologie, Institut Pasteur de Bangui, Bangui, République Centrafricaine; 5 Institut de Santé Publique Epidémiologie Développement (ISPED), Université de Bordeaux, Bordeaux, France; 6 Centre Inserm, Université de Bordeaux, Bordeaux, France; Hôpital Bichat-Claude Bernard, FRANCE

## Abstract

Human papillomavirus (HPV) infection is the most common sexually transmitted disease worldwide, and it particularly affects people living with human immunodeficiency virus (HIV). This study aimed to estimate the prevalence of HPV and to describe HPV genotypes in HIV-1 infected women in Lomé, Togo. From September 2014 to September 2015, a cross-sectional study was conducted in two treatment and care centers for people living with HIV: the Centre Hospitalier Universitaire Sylvanus Olympio and the non-profit organization ‘Espoir Vie Togo’. Women living with HIV-1, aged 18 years and older, receiving a combination antiretroviral therapy for at least 12 months, and who gave their informed consent to participate in the study were recruited. Cervical swabs were collected using a cytobrush, and cells were stored in a preservative solution. HPV testing was performed using e-BRID equipment. Blood samples were collected for CD4+ count using a flow cytometer and for HIV viral load using polymerase chain reaction. A total of 221 HIV-1 infected women were enrolled. The prevalence of any type and oncogenic HPV was 22.2%, 95% confidence interval (95% CI): [17.1–28.2] and 16.7% (95%CI: 12.3–22.3), respectively. The most prevalent genotypes were: 18 (8.6%), 68 (4.1%), and 62/81 (2.7%). Only 1.3% (3/221) of participants were infected with HPV16. In regression analysis, no factor was associated with HRHPV.

**Conclusion:**

This study showed the diversity of circulating HPV genotypes in Togo. Programs of HPV vaccination and early detection of benign or precancerous lesions should be implemented to reduce cancer-related comorbidities.

## Introduction

Human Papillomavirus (HPV) infection is the most common sexually transmitted virus worldwide [1, 2]. HPV are grouped into oncogenic or high-risk HPV (HR-HPV) (16, 18, 31, 33, 35, 39, 45, 51, 52, 56, 58, 59, 69) and non-oncogenic or low risk HPV (LR-HPV) (6, 11, 42, 43, 44 and 53) [3–5]. The oncogenicity of HR-HPV is essentially based on two viral oncoproteins with transforming properties, called E6 and E7, which can interact with the products of tumor-suppressor genes p53 and prb [6–8]. HPV infection is associated with cervical cancer in women. Despite the introduction of cervical cancer screening programs, approximately 528,000 new cases and 266,000 deaths occur each year worldwide with 85% of deaths occurring in developing countries [1, 9]. HPV is also involved in many skin and mucosal cancers. The virus, which has a mucous tropism, is transmitted more particularly but not exclusively by sexual means [10, 11]. One in five women with normal cervical cytology is reported to be infected with HPV in sub-Saharan Africa, which is also the most affected region by Human Immunodeficiency Virus (HIV) infection [12]. Co-infection with HIV infection is a factor facilitating carcinogenesis associated with HR-HPV infections. Prospective studies have reported a higher incidence of HPV among HIV-positive women compared to HIV-negative women [1, 13–15]. In Côte d’Ivoire, in 2012, out of 445 women of which 254 were HIV-positive, the prevalence of HR-HPV infection was 53.9% in HIV-positive women compared to 33.7% in HIV-negative women. Nowadays, the extent of cervical cancer and HPV infection can be reduced, and control strategies rely on HPV vaccination and early detection of benign or precancerous lesions [16]. In Togo where cervical cancer is a public health problem, it is the second most common cancer in women [17], with an estimated mortality rate of 12.8% [18]. However, limited data are available on circulating genotypes in the country, especially among HIV-infected women while HPV vaccination recommendations for people living with HIV (PLWHIV) are under consideration. The objective of this study was to estimate the prevalence of HPV infection and to describe the distribution of circulating genotypes in HIV-1 infected women in Lome, Togo.

## Materials and methods

### Study design and setting

A cross-sectional study was carried out over a period of 13 months (from September 2014 to September 2015) in two leading treatment and care centers for PLWHIV in Lomé: the Centre Hospitalier Universitaire Sylvanus Olympio (teaching hospital) and the non-profit organization ‘Espoir Vie Togo’.

### Sample size and participants

Women living with HIV-1, aged 18 years and older, receiving a combination antiretroviral therapy (cART) for at least 12 months, and who gave their informed consent to participate in the study were recruited. The first-line treatment included two nucleoside reverse transcriptase inhibitors (NRTIs), Lamivudine (3TC) + Zidovudine (AZT) combined with a non-nucleoside reverse transcriptase inhibitor (NNRTI), Efavirenz (EFV) or Nevirapine (NVP).

Since no data on HPV infection were available in Togo, the sample size calculation was based on the following assumptions: an expected prevalence of HPV infection in HIV infected women of 60% in Burkina Faso [19], neighboring country of Togo, with a precision of 7% and a significance level set at 5%; the minimum sample size was estimated at 188 participants.

### Data collection

A standardized questionnaire was used in a face-to-face interview to record participants’ socio-demographic characteristics and medical history.

A cervical swab was obtained using a cytobrush to collect cells at the junction area between the endocervix and the exocervix. Cells were then collected in a preservative solution (Cytofast solution 42010600, Hospitex Diagnostics S.r.l.). The sample was transported and stored at room temperature (10–30°C) for two to five days before manipulation at the Laboratoire de Biologie Moléculaire et Immunologie (BIOLIM) of the Faculté des Sciences de la Santé of the Université de Lomé (Faculty of Health Sciences at the University of Lomé).

Two blood samples were drawn for counting CD4+ T-cell and measuring HIV viral load. These samples were transferred to the BIOLIM laboratory at 4°C within 4 hours. CD4+ count was performed immediately, then plasma was collected after centrifugation on the second tube, aliquoted, and stored at -80°C for HIV viral load measurement. Women living with HIV and having an HIV viral load greater than or equal to 10 000 copies / mm^3^ after one year of treatment were described as having inadequate HIV virological control.

### HPV testing, HIV viral load detection, and CD4+ count

Screening for HPV infection was performed after amplification by Polymerase Chain Reaction (PCR) and hybridization of HPV deoxyribonucleic acid (DNA) molecules. The PCR Mix and Phire Hot Start II DNA Polymerase (MAD-003930MU-P-E-30, Master Diagnostica) kit was used for amplification. The resulting PCR product was then hybridized using the optimized HPV Direct Flow CHIP kit (MAD-003930M-H, Master Diagnostica) following the protocol provided by the manufacturer on the controller (e-BRID System of Hospitex Diagnostics). This system allows screening and genotyping of 18 HR-HPV (16, 18, 26, 31, 33, 35, 39, 45, 51, 52, 53, 56, 58, 59, 66, 68, 73 and 82) and 18 LR-HPV (6, 11, 40, 42, 43, 44, 54, 55, 61, 62, 67, 69, 70, 71, 72, 81, 84 and CP6108) [20]. The HIV plasma viral load was carried out using Abbott Real-Time HIV-1 VL quantitative RT-PCR assays (Abbott molecular, IL, USA), and a manual extraction system coupled with a m2000rt amplification and detection system. The limit of detection was < 40 copies/mL [21, 22]. CD4+ count was performed on a flow cytometer, Facscalibur (BD, Sciences, Franklin Lakes, NJ USA 07417) in all patients.

### Statistical analyzes

The data collected were cleaned, coded, and entered into a Microsoft Excel database developed for this purpose. Data analysis was performed using STATA software version 14.1 (StataCorp, College Station, Texas, USA). Results were presented as proportions for categorical variables and as medians for quantitative variables. The prevalence of HPV infection, for example, has been presented with its 95% confidence interval. Comparison of categorical variables was performed with the Chi2 test or the exact Fisher test, and comparison of quantitative variables was carried out with Student's t-test or the analysis of variance test. Regression analyses were performed to identify factors associated with HRHPV infection. The significance was set at 5%.

### Ethical considerations

The study is a project of the Ministry of Health of Togo. It was approved by the “Comité de Bioéthique pour la Recherche en Santé (CBRS)” (Bioethics Committee for Health Research) from the Ministry of Health of Togo (n°751/2014/MS/CAB/DGS/DPLET/CBRS). Participants provided written consent prior to participation and authorizations from the Directors of selected medical centers were obtained before conducting the study.

## Results

### Sociodemographic, clinical and biological characteristics of the participants

A total of 221 HIV-1 women taking cART were enrolled. The median age of study participants was 36 years old, interquartile range (IQR): [29–45]. The majority (79.6%) had at least a secondary education, and 50.7% were living with a partner. The median CD4+ count was 346 cells / mm3 (IQR: [233–456]), and the majority (76.5%) of HIV-infected women had a CD4+ count less than 500 cells / mm^3^. HIV viral load was greater than or equal to 10 000 copies / mm^3^ in 57.9% of women living with HIV (Table 1).

**Table 1:** HPV prevalence according to sociodemographic and clinical characteristics.

Characteristics	Total (n = 221)	*HPV+ (n = 49)			**HRHPV+ (n = 37)			***LRHPV+ (n = 15)		
	N	n	(%)	P	N	(%)	P	n	(%)	P
**Age (years)**				0.136			**0.044**			0.981
20–29	57	12	(21.1)		10	(17.5)		4	(7.0)	
30–39	70	13	(18.6)		11	(15.7)		4	(5.7)	
40–49	59	11	(18.6)		5	(8.5)		5	(8.5)	
≥ 50	35	13	(37.1)		11	(31.4)		2	(5.7)	
**Marital status**				0.705			1.000			0.748
Lives alone	109	23	(31.1)		18	(16.5)		8	(7.3)	
In a relationship	112	26	(23.2)		19	(17)		7	(6.3)	
**Education level**				0.307			0.794			
None or primary	45	11	(24.4)		9	(20.0)		3	(6.7)	0.738
Secondary	97	25	(25.8)		15	(15.5)		8	(8.2)	
University	79	13	(16.5)		13	(16.5)		4	(5.1)	
**Economic situation**				0.759			0.461			0.741
IGA†	177	40	(22.6)		28	(15.8)		13	(7.3)	
No IGA	44	9	(20.5)		9	(20.5)		2	(4.5)	
**Number of pregnancies**				0.432			0.742			0.938
< 2	101	20	(19.8)		16	(15.8)		7	(6.9)	
≥ 2	120	29	(24.2)		21	(17.5)		8	(6.7)	
**HIV viral load (copies/ mm**^**3**^**)**				**0.017**			0.208			**0.015**
< 10 000	93	14	(15.1)		9	(9.7)		6	(6.4)	
≥ 10 000	128	35	(27.3)		28	(21.9)		9	(7.0)	
**CD4 count (cells/mm**^**3**^**)**				0.559			0.469			0.354
< 500	169	39	(23.1)		30	(17.8)		10	(5.9)	
≥ 500	52	10	(19.2)		7	(13.5)		5	(9.6)	

†IGA: Income generating activities;

*HPV +: women infected with HPV;

**HRHPV+: women infected with HRHPV;

***LRHPV: Low-risk HPV

#### Prevalence of HPV infection

The prevalence of HPV infection was 22.2%, 95% confidence interval (95% CI): [17.1–28.2]. HPV infection was not associated with age (p = 0.136). More than one-third (37.1%) of women aged 50 years and older, and 17.5% of women aged 20 to 29 had HPV infection. The remaining sociodemographic factors were not associated with HPV infection (p>0.05). Only inadequate HIV virological control was associated with HPV infection (p = 0.017) (Table 1).

The prevalence of HR-HPV was 16.7% (95% CI: 12.3–22.3). This prevalence was significantly higher among women aged 20 to 29 years (10/57; 17.5%), 30 to 39 years (11/70; 15.7%), and those over 50 years (11/35; 31.4%) (p = 0.04) (Table 1). Among HPV infected women, HR-HPV was observed in 75.5% (37/49) and LR-HPV in 30.6% (15/49). Unclassified genotypes were found in three women (3/49; 6.1%). HR-HPV was found mainly in women who had at least two children (21/120; 17.5%), had none or primary level education (9/45; 20.0%), and with no income-generating activities (9/44; 20.5%). However, these findings were not statistically significant (p>0.05) (Table 1).

### HPV genotypes

The most prevalent genotypes were: 18 (8.6%), 68 (4.1%), and 62/81 (2.7%). Only three (1.3%) women were infected with HPV16. Single and multiples infections were observed in 71.4% (35/49) and 28.6% (14/49) of HPV infected participants, respectively. Of the 37 HR-HPV infected women, 78.4% (29/37) had a single infection and 21.6% (8/37) had multiple infection. Among LR-HPV infected women, 60.0% (9/15) had a single infection and 40.0% (6/15) had multiple infection. Of the eight identified oncogenic strains, the Cervarix and Gardasil-9 vaccines covered two strains for 64.9% and four strains for 75.7% of the participants, respectively.

**Fig 1:**
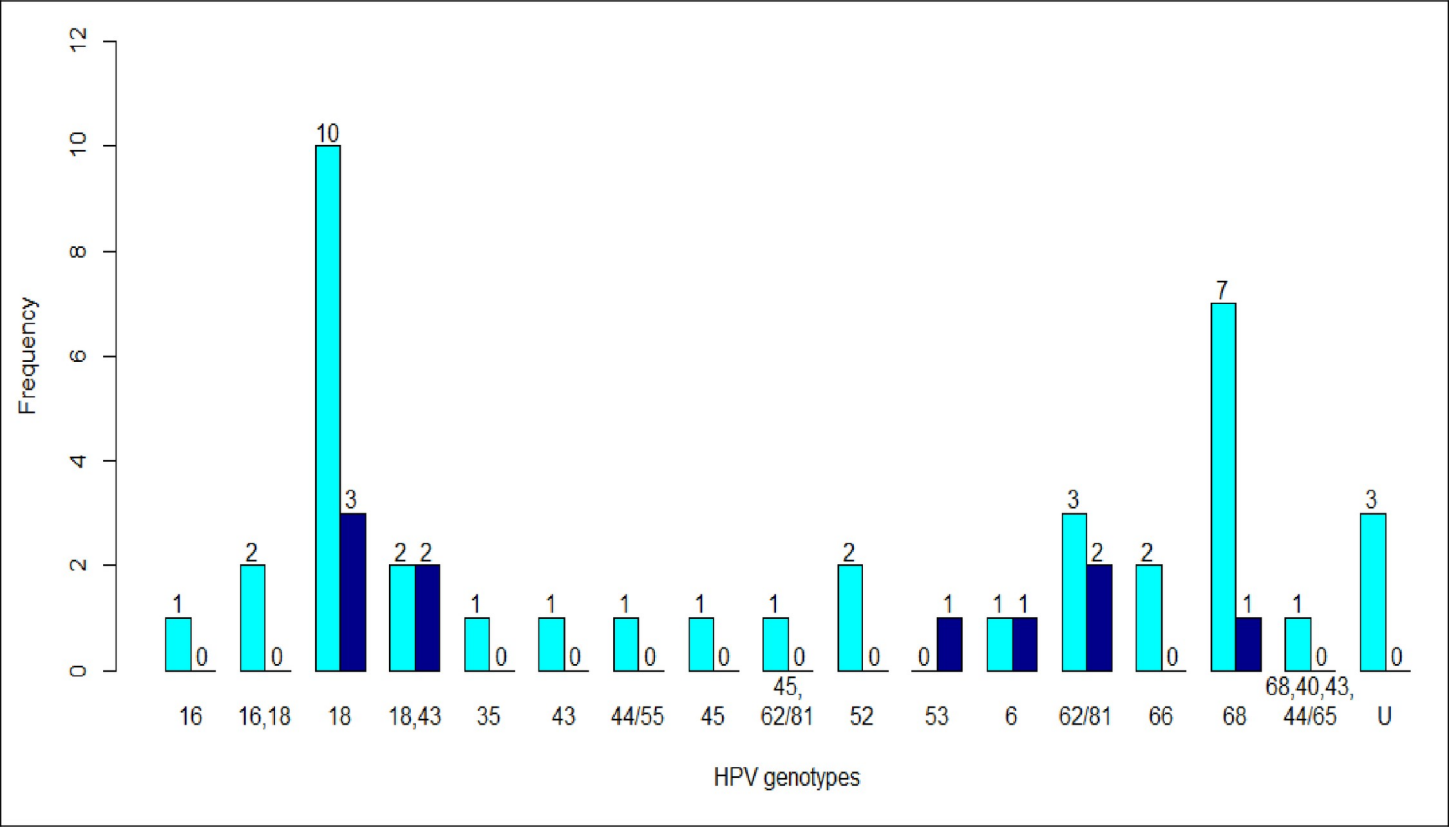
HPV genotypes according to CD4+. The frequency of each HPV genotype was presented in light blue bars and dark blue for women with CD4+count less than 500 cells/mm^3^ and greather than 500 cells/mm^3^, respectively.

**Fig 2:**
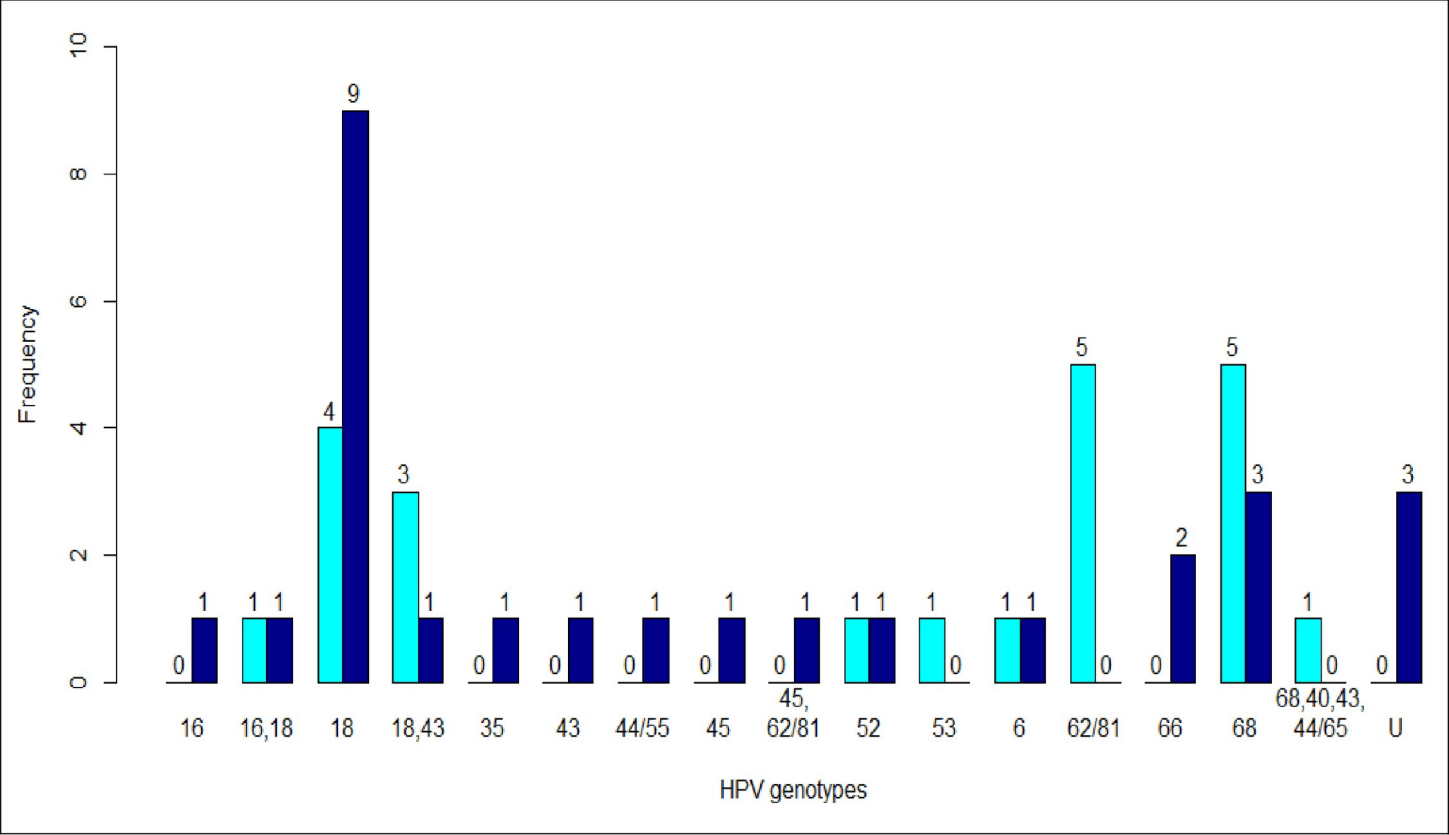
HPV genotypes according to HIV viral load. The frequency of each HPV genotype was presented in light blue bars and dark blue for women with HIV viral load less than 10 000 copies/mm^3^ and greather than 10 000 copies/mm^3^, respectively.

Genotypes 18, 68, and 62/81 were more frequently identified in women with CD4+ count below 500 cells/mm3 (p>0.05) (Fig 1). Among women who had inadequate HIV virological control, HPV 18 was more frequently identified (p = 0.0015) (Fig 2).

### Factors associated with HPV infection

In univariate analyses, none of the explanatory variables were associated with HRHPV. Therefore, multivariate analyses were not conducted (Table 2).

**Table 2:** Factors associated with HRHPV infection.

	HRHPV infection				Univariate Analysis		
	No		Yes		OR	95%CI	p value
	N	%	N	%			
**Age (years)**							0.9724
< 35	79	42.9	16	43.2	1		
≥ 35	105	57.1	21	56.8	0.98	[0.48–2.01]	
**Marital status**							0.9286
Lives alone	91	49.5	18	48.7	1		
In a relationship	93	50.5	19	51.3	1.03	[0.51–2.09]	
**University level**							0.9324
Yes	66	35.9	13	35.1	1		
No	118	64.1	24	64.9	1.03	[0.49–2.16]	
**Economic situation**							0.4622
IGA†	149	81	28	75.7	1		
No IGA	35	19	9	24.3	1.36	[0.59–3.15]	
**Number of pregnancies**							0.7423
< 2	85	46.2	16	43.2	1		
≥ 2	99	53.8	21	56.8	1.12	[0.55–2.29]	
**HIV viral load (copies/mm**^**3**^**)**							0.8754
< 10 000	77	41.9	16	43.2	1		
≥ 10 000	107	58.1	21	56.8	0.94	[0.46–1.92]	
**CD4 count (cells/mm**^**3**^**)**							0.4704
≥ 500	45	24.5	7	18.9	1		
< 500	139	75.5	30	81.1	1.38	[0.57–3.37]	

†IGA: Income generating activities;

HPV: Human papillomavirus; OR: Odds ratio

## Discussion

This study is one of the firsts conducted on HPV infection in Togo where cervical cancer is the second leading cause of cancer related death in women after breast cancer [17]. Other studies are being conducted among female sex workers and men who have sex with men in order to have a detailed mapping of HPV strains in Togo.

HPV prevalence was estimated at 22.2%. This prevalence is one of the lowest reported in sub-Saharan Africa. According to a meta-analysis by Clifford et al. in 2006, the prevalence of HPV infection was estimated at 56.6% in Africa, 31.1% in Asia, 32.4% in Europe, 31.4% in North America, and 57.3% in Central and South America among women living with HIV [23]. In the West African region, particularly in Côte d’Ivoire and Senegal, the HPV prevalence was two to three times higher than that found in our study among women living with HIV and under cART [16, 24]. In our study, none of the sociodemographic data were associated with HPV prevalence in contrast with that observed in Côte d’Ivoire [16]. Several reasons can explain the low HPV prevalence observed in Togo compared to results obtained in neighboring countries such as the proportion of women on cART or with virological suppression. In Côte d’Ivoire, only 74.8% of HIV infected women were on cART [16]; whereas, in our study all women were under treatment. In Burkina Faso, this proportion was not specified [19]. Conducting behavioral studies in Togo, including condom use, number and type of sexual partners, may provide more insights about this low prevalence.

Despite the low HPV prevalence, oncogenic strains remained more prevalent as reported in other studies in the general population or in PLWHIV [12, 25–27]. The genotype 16 prevalence was low in our study but was mostly found in other studies in the general population [28–30]. In the case of immunosuppression, in PLWHIV or in transplant patients, there is an apparent decrease in the prevalence of genotype 16 in favor of other types whose oncogenic power would be promoted by immunosuppression [31]. In addition, multiple infections were observed in 28.6% of women, as reported by Jaquet et al, who found 24% of multiple HPV infections in Côte d’Ivoire [16]. These multiple infections constitute a huge challenge for optimal vaccine coverage against HPV infection.

The distribution of the majority of genotypes differs greatly from one geographical area to another, with genotypes 35 (15.7%), 16 (14.2%), 18 (11.4%) and 58 (11.4%) in Côte d’Ivoire; 16 (30.4%), 35 (20.3%), 53 (19.6%) and 18 (18.2%) in South Africa and 6 (40%), 40 (14%), 16 (12%), and 52 (9%) in Tunisia [16, 25, 32]. With the variability of the distribution of genotypes from one country to another, it is essential to have data from each region in order to draft sound and meaningful HPV vaccination policies for the general population and PLWHIV. Early detection of HPV infections in HIV-infected women is urgently needed to better organize their management [32, 33]. The cervical smear is a good screening tool for women who are HIV-positive, so there is no need for systematic colposcopy [34]. For the general population, the combination of smear and oncogenic HPV testing for the diagnosis of cervical lesions appears to be a promising tool. HPV Direct Flow CHIP e-BRID) used in this study, is intended for simultaneous detection and genotyping of 36 HPV types (18 HR and 18 LR) by PCR followed by reverse dot blot automatic hybridization, based on DNA-flow Technology with the automatic-BRID System. Clinical samples can be amplified directly without having to extract them. Other tests exist as Linear Array and INNO-LiPA. Linear Array is a qualitative strip test that identifies 37 HPV genotypes (15HR, 3 HPV potentially HR and 19 LR). The INNO-LiPA test is also a strip test that detects 28 HPV genotypes (18 HR, 7 LR and 3 HPV unclassified). The identified genotypes of these tests are determined by comparison with a reference strip. HPV Direct Flow CHIP has the advantage of analyzing large series of samples and makes reading easier compared to tests using strips. In addition, it is faster to perform than strip tests, 90 minutes after DNA amplification is required to obtain results with HPV Direct Flow CHIP test against 8 hours with the INNO-LiPA test [20].

The HPV and HR-HPV prevalence were higher after 50 years, and there was a statistically significant difference according to age. Similar results were reported in a meta-analysis by Bruni et al., where a peak occurred after 45 years in South and Central America and after 55 years in West Africa [12]. In Côte d’Ivoire, an ascension of HR-HPV infection was observed among HIV infected women after age of 50 [16]. Senescence of cellular immunity and immunosuppression may be involved in these reactivations, explaining the peak of infection observed in postmenopausal women or the recurrence of infection in HIV-infected persons. Other hypotheses have been put forward to explain the peak in 50’s in particular, the persistence of HPV, the changes of sexual partners at the age of 50, the cohort effect or the lack of organized screening for cervical cancer [12]. For the latter possibility, in regions such as Europe and Northern America where effective screening is carried out among women aged less than 40 years, it has been observed that the second peak is attenuated [12]. Similar trends have been reported in Costa Rica where the U-shaped curve of age-specific HPV prevalence, observed at enrollment, became less pronounced at follow-up [35]. In fact, cervical cytology not only reduces precancerous lesions and HPV related persistent infections, but removing these lesions may have a direct antigen-presenting effect that could protect against subsequent HPV infections [36]. Regular screening procedures suitable for women aged 50 years and over must be implemented in the current context of the ageing world population, and longer life expectancy of PLWHIV with antiretrovirals.

Genital HPV infections can be prevented by vaccination. The prerequisite for this vaccination is the knowledge of the molecular epidemiology of circulating HR-HPV in a given region. Two vaccines are currently marketed: Gardasil-9, which protects against genotypes 6, 11, 16, 18, 31, 33, 45, 52, 58, and Cervarix, which protects against infections caused by genotypes 16 and 18. The WHO recommends that the vaccine be administered to young adolescents aged 9 to 13 years, and a three-dose regimen is recommended for immunodeficient persons, including PLWHIV and all young women under 15 years old [10, 37]. In Togo, there is no HPV vaccination schedule that has been validated and recommended by the Ministry of Health. Based on the results of this study, Cervarix and Gardasil-9 cover HR-HPV genotypes which circulate among the majority of HIV-infected women in Togo with better coverage for Gardasil-9. With the morbidity and mortality of cervical cancer being very high in Togo, it is urgent to establish national anti-HPV vaccine recommendations and to advocate for improved access to these vaccines for target populations.

The persistence of HPV infection depends on viral factors such as HIV viral load, HPV DNA integration, regulatory protein (E6 and E7), and field-related characteristics (immune, genetic, cocarcinogen response). In the cervix, the majority of HPV is spontaneously eliminated within one to two years. This immune response is often moderate and delayed, leading to the onset and persistence of the infection. Immunosuppression is strongly associated with the development of HPV infection [16, 24]. The HIV viral load is a key factor for therapeutic follow-up and prevention of co-infections like HPV infection. The effects of cART on chronic infection and the risk of precancerous lesions and cancer are important in women with HR-HPV. In our study, HPV infection was significantly higher in women who had HIV viral load greater than or equal to 10 000 copies/mm^3^. There is a real problem of compliance and care and Togo has been identified as a country with high a rate of resistance mutations [38–40]. To resolve this matter, efforts including strengthening the capacity of medical practitioners on routine HIV viral load measurements are needed, as well as, creating therapeutic education clubs and switching to second line regimen.

This study had several limitations with regards to study design and biological procedures. First, recruitment was difficult because of the low acceptability of cervico-vaginal specimens, hence a possible selection bias should not be excluded. Also, the generalizability of findings of this hospital-based study should be done with caution, since women recruited in our study could be different from those in the community, leading to an underestimation or overestimation of HPV prevalence. Finally, since we conducted a cross-sectional study, we could not infer causality between HPV and HIV as we did not record incident HPV cases among HIV infected women. However, with this study we were able to describe the diversity of HPV genotypes circulating in HIV-infected women.

Some biological samples were unusable due to the lack of sufficient biological material for amplification and genotyping of the virus. The technique used in this study is simple, fast, and less expensive but needs to be improved. Washing the cells in the buffer had a limit for some samples that could not be amplified and genotyped. Prior extraction of the virus DNA from these samples or a complexation reaction of the inhibitors would allow its optimization. Also, the analysis of cytopathological data was not performed in this study to confirm or refute the presence of precancerous and cancerous lesions of the cervix. In Togo, despite existing national recommendations, visual inspection with Lugol’s iodine (VILI) and visual inspection with acetic acid (VIA) are not yet available in HIV care settings. Women screened positive for HPV were referred for colposcopy and gynecology for follow-up as recommended by national guidelines.

## Conclusion

HPV prevalence was 22.2% among HIV infected women in Togo. HR-HPV genotypes 18 and 68 were more common in women with HIV viral load greater than or equal to 10 000 copies/mm^3^. HPV vaccination and early detection of HPV infection in women living with HIV would reduce this comorbidity. Behavioral, cytological and even cohort studies are required in order to identify factors associated with the HPV low prevalence. In addition, it will be necessary to intensify the sensitization and to integrate the cervical cancer screening into routine practices.

## Supporting information

S1 File Database Togo HIV-HPV 2015. (XLSX)

S2 File Research protocols Togo HIV-HPV. (DOCX)
