# Supplementary material for: Prevalence and distribution of Human Papillomavirus (HPV) genotypes among HIV infected women in Lomé, Togo
Source: PLoS One. 2019 Feb 27;14(2):e0212516. doi: 10.1371/journal.pone.0212516 (PMC6392291; doi:10.1371/journal.pone.0212516)
Supplement: S2 File — (DOCX) [file pone.0212516.s002.docx]

**PROTOCOLE DE RECHERCHE SUR LE THEME**

**PREVALENCE ET DIVERSITE GENETIQUE DU PAPILLOMAVIRUS HUMAIN (VPH) ET CO-INFECTION VPH - VIH AU TOGO**

**_______________________________________________________________**

Protocole de recherche en vue de l’obtention du doctorat du 3^e^ cycle de la Faculté des Sciences de l’Université de Lomé

**Présenté par NYASENU Tufa Yawo**

**Directeur :**

**Professeur Agrégé Anoumou Yaotsè DAGNRA**

**Faculté des Sciences de la Santé**

**Université de Lomé**

**Année : 2015**

**Sommaire**

1. **Contexte et justification**
2. **Objectif**
3. **Méthode**
   1. **Schéma d’étude**
   2. **Sites de recrutement des participants**
   3. **Population d’étude**
   4. **Taille de l’échantillon**
   5. **Recueil des données**
   6. **Analyses de laboratoires**
   7. **Variables à étudier**
   8. **Traitement des données**
      1. **Saisie des données**
      2. **Analyse des données**
   9. **Aspects éthiques**
4. **Chronogramme**
5. **Archivage**

**Références**

**Annexes**

1. **Contexte et justification**

L’association entre le cancer du col et le Virus du Papillome Humain (VPH) est bien connue puisque le VPH a été reconnu comme étant impliqué dans 99,7% des cas de cancers du col. De nombreuses études ont mis en évidence une prévalence élevée de la coinfection à VPH avec l’infection au Virus de l’Immunodéficience Humaine (VIH), avec une augmentation à la fois des infections à VPH latentes et des infections symptomatiques. Des études prospectives utilisant la PCR (PolymeraseChain Reaction) pour détecter le VPH, ont retrouvé une incidence de 95% chez les femmes VIH-positives, contre 22% chez les femmes VIH-négatives [1,2].

Le cancer du col est la deuxième cause de cancers féminins dans le monde et représente environ 10 % de la totalité des cancers. Le nombre de cancers invasifs était estimé à 493 000, 83 % de ces cancers étant observés dans les pays en voie de développement [3]. Les zones à risque pour le cancer du col sont situées en Afrique du Sud et de l’Est, aux Caraïbes et en Amérique centrale où l’incidence moyenne est supérieure à 30 pour 100 000 femmes par an. Chaque année, on estime à 273 000 les décès induits dont trois quarts sont enregistrés dans les pays en voie de développement [4]. C’est la première cause de décès par cancer chez la femme dans les pays en voie de développement et la 10ème cause de décès dans les pays développés. En France, il occupe le 8ème rang des cancers féminins (3390 nouveaux cas) et le 5ème rang en termes de mortalité (1160 décès) [5,6].

Le cancer du col utérin est l’un des rares cancers humains évitables. En effet, sa prévention repose sur le diagnostic très précoce des lésions bénignes ou précancéreuses dont le traitement rend en principe impossible le développement d’un cancer. Fondée sur la pratique du frottis qui consiste à prélever les cellules du col, l’analyse morphologique des modifications de ces cellules est suivie de la réalisation d’un examen plus précis, la colposcopie, qui localise les anomalies à la surface de l’épithélium cervical. Le diagnostic et le traitement qui s’ensuivent, permettent, en théorie, d’éviter le développement d’un cancer invasif [7,8]. Cette démarche, qui va du dépistage à la prévention, est unique pour le site du col utérin.

Parmi les maladies dues au VPH, on peut citer les cancers du col utérin, du vagin, de la vulve, du pénis et de l’anus ; un sous-groupe de cancers de la tête et du cou ; des condylomes anogénitaux et une papillomatose respiratoire récurrente. En 2005, il y a eu dans le monde près de 500 000 cas de cancer du col utérin et 260 000 décès associés. L’incidence du cancer du col se situe entre 1 et 50/100 000 femmes ; c’est en Amérique latine et aux Caraïbes, en Afrique subsaharienne, en Mélanésie, dans le sud de l’Asie centrale et en Asie du Sud-Est qu’elle est la plus élevée. La plupart des cas du cancer du col sont diagnostiqués chez des femmes âgées de plus de 40 ans [9]. L’Afrique sub-saharienne, reste aussi la région la plus touchée par l’infection à VIH depuis sa découverte il y a trente ans. L’épidémie du SIDA demeure toujours un problème de santé publique. En 2012, le nombre de personnes infectées par le VIH est estimé à 35,3 millions et l’infection est à l’origine de près de 2 millions de décès par an selon le rapport de l’ONUSIDA [10]. Près de 2/3 des personnes vivant avec le VIH (PVVIH) se retrouvent en Afrique sub-saharienne.

Dans la perspective d’atteindre l’objectif mondial « zéro nouvelle infection à VIH, zéro discrimination et zéro décès lié au sida », le monde est en passe de fournir une thérapie antirétrovirale à 15 millions de personnes d’ici 2015. En 2012 ; 9,7 millions de personnes ont suivi une thérapie antirétrovirale dans les pays à revenu faible et intermédiaire, soit 61 % des personnes éligibles selon les directives de 2010 sur le VIH de l’Organisation mondiale de la Santé (OMS). Entre 1996 et 2012, la thérapie antirétrovirale a permis d’éviter 6,6 millions de décès liés au sida dans le monde, dont 5,5 millions dans les pays à revenu faible et intermédiaire. L’élargissement de l’accès aux traitements antirétroviraux (TAR) contribue nettement à la baisse, chaque année, du nombre de nouvelles infections à VIH dans le monde, y compris chez l’enfant. L’extension des programmes de PTME et l’utilisation de schémas thérapeutiques plus efficaces ont permis de prévenir plus de 800 000 infections chez l’enfant entre 2005 et fin 2012. Sur 90 % des femmes enceintes qui vivent avec le VIH et où surviennent 90 % des nouvelles infections chez l’enfant, le taux de transmission mère-enfant, est passé globalement de 26% [24-30%] en 2009 à 17% [15-20%] en 2012 [11]. L’accès élargi aux TAR modifie profondément l’épidémie de l’infection à VIH dans le monde. Les taux de mortalité liée au SIDA baissent rapidement ; 4,2 millions de décès dans les pays à revenu faible ou intermédiaire ont été évités de 2002 à 2012.

Les efforts de prévention continuent de porter leurs fruits, comme en témoigne le nombre de nouvelles infections à VIH chez les adultes dans les pays à revenu faible et intermédiaire qui était de 1,9 million en 2012, soit une baisse de 30 % par rapport à 2001. En 2013, le Togo figurait dans les 26 pays où l’incidence du VIH chez les adultes a baissé de plus de 50 % entre 2001 et 2012. La baisse du taux de nouvelles infections à VIH dans ses 26 pays à revenu faible et intermédiaire atteste ces efforts [10]. Les interventions de lutte contre la co-infection tuberculose-VIH ont permis de sauver plus de 400 000 vies en 2011 seulement (huit fois plus qu’en 2005). Mais à l’heure actuelle, les données sur la co-infection VPH-VIH sont rares malgré que ces deux infections sévissent plus en Afrique subsaharienne.

L’infection à VPH pourrait s’avérer problématique pour les personnes atteintes du VIH/sida, puisque les ressources limitées du système immunitaire sont compromises lors de la présence d’une infection virale ou d’un cancer. La coïnfection à VPH et à VIH peut entraver les défenses immunitaires contre certaines complications telles que le cancer anal et celui du col utérin.

Les infections génitales à VPH comme l’infection à VIH sont principalement transmises par contact sexuel, le plus souvent mais pas exclusivement lors d’un rapport avec pénétration. Les VPH sont hautement transmissibles et la plupart des hommes et des femmes sexuellement actifs contracteront une infection à VPH à un moment ou à un autre de leur vie. Tandis que la plupart des infections à VPH sont transitoires et bénignes, une infection génitale persistante par certains génotypes viraux peut conduire au développement de lésions précancéreuses et de cancers ano-génitaux.

Il existe plus de 100 génotypes VPH. Certains sont associés à une immortalisation et à une transformation des cellules liées à la cancérogenèse. Parmi eux, au moins 13 sont susceptibles de provoquer un cancer du col ou sont associés à d’autres cancers ano-génitaux et oropharyngiens. Les VPH de type 16 et 18 sont à l’origine de près de 70% de tous les cas de cancer du col invasif dans le monde, le type 16 ayant le potentiel oncogène le plus important. La répartition des types de VPH varie selon les régions géographiques, mais le type oncogène dominant dans l’ensemble des régions est le VPH-16 [12]. A l’heure actuelle, 2 vaccins anti-VPH sont largement commercialisés au plan international. Ils sont uniquement destinés à l’usage prophylactique ; ils n’éliminent pas une infection existante ni ne traitent une maladie liée au VPH [13]. Ces deux vaccins sont destinés à l’administration chez la jeune fille (10 à 14 ans) avant le début de l’activité sexuelle, c’est-à-dire avant la première exposition à l’infection par le VPH. En juin 2007, Le Groupe stratégique consultatif d’experts (SAGE) qui est le principal groupe consultatif de l’OMS pour les vaccins et la vaccination a conclu que ces deux vaccins avaient de bons profils d’innocuité [14]. L’OMS reconnaît l’importance du cancer du col utérin et des autres maladies liées à VPH en tant que problèmes de santé mondiaux et recommande d’inclure la vaccination systématique contre le VPH dans les programmes nationaux de vaccination pour autant que : la prévention du cancer du col utérin ou des autres maladies liées au PVH, ou les deux, soit une priorité de santé publique.

Il s’avère indispensable pour le Togo, d’évaluer la prévalence et la diversité génétique des VPH circulants chez les personnes infectées par le VIH, parce que la persistance de l’infection et sa tumorigénécité est très variable ; puis pour mieux apprécier l’efficacité et l’indication des vaccins disponibles.

Ce travail est une contribution pour l’étude de la prévalence et de la diversité génétique du VPH et pour l’optimisation de la prise en charge des PVVIH infectés par le VPH.

1. **Objectif**

**2.1** **Objectif général**

Etudier la distribution du HPV chez les PVVIH.

**2.2** **Objectifs spécifiques**

- Estimer la prévalence de l’infection du Virus du Papillome Humain (VPH) chez les femmes vivant avec le VIH ;
- Déterminer la distribution des types circulants du Virus du Papillome Humain ;
- Estimer la prévalence de la co-infection VIH/VPH

1. **Méthodes**

**3.1 Schéma d’étude**

Il s’agira d’une étude transversale à visée descriptive et analytique qui se déroulera du 1^er^ Septembre 2014 au 30 Septembre 2015.

**3.2 Site de recrutement des participants**

Les participants seront recrutés dans deux grands centres de soins médicaux pour les PVVIH à Lomé, notamment :

- le Centre Hospitalier Universitaire Sylvanus- Olympio : CHU-SO

- et une structure associative, Espoir Vie Togo : EVT

- 1. **Population d’étude**

Critères d’inclusion du recrutement :

Le recrutement des femmes et jeunes filles se fera selon les critères d’inclusion suivants :

- Patient de sexe féminin,
- Âgée de 18 ans et plus,
- Sous traitement antirétroviral (TAR) de première ligne depuis au moins 12 mois : le traitement de première ligne comporterait deux inhibiteurs nucléosidiques de la transcriptase inverse (INTI), Lamivudine (3TC) + Zidovudine (AZT) associés à un inhibiteur non nucléosidique de la transcriptase inverse (INNTI), Efavirenz (EFV) ou Névirapine (NVP).
- Ayant signé une fiche de consentement.

Critères de non inclusion

- Patientes nouvellement dépistées pour l’infection à VIH
- Patientes non mises sous TAR
- Patientes connues vivant avec le cancer du col de l’utérus
  1. **Taille de l’échantillon**

Pour le calcul de la taille de l’échantillon, la formule suivante a été utilisée :

$$n\geq\frac{u^{2}\pi( 1-\pi)}{\Delta^{2}}$$

Où :

- u = 1,96 au seuil de 5%
- π = 0,60 le taux de couverture vaccinale
- ∆= 0,04 la précision

Comme aucune donnée sur l'infection à VPH n'était disponible au Togo, le calcul de la taille de l'échantillon a été basé sur les hypothèses suivantes : une prévalence attendue de l'infection à VPH chez les femmes infectées par le VIH de 60% au Burkina Faso [15], pays voisin du Togo, avec une précision de 7% et un seuil de significativité fixé à 5% ; la taille minimale de l'échantillon a été estimée à 188 participants.

- 1. **Recueil des données**
     1. Questionnaire

Un questionnaire standardisé et préalablement testé, sera administré en face à face aux participantes pour le recueil des données sociodémographiques, les antécédents médicaux et les modalités de prise en charge (voir fiche d’enquête).

- - 1. Prélèvement
- Prélèvement vaginal

Le frottis de dépistage cervico-vaginal sera réalisé à l’aide d’une cytobrosse pour prélever les cellules au niveau de la zone de jonction de l’endocol et de l’exocol. Les cellules ont ensuite été recueillies dans la solution Cyofast de conservation (42010600, Hospitex Diagnostics S.r.l.). L’échantillon a été acheminé et stocké à la température ambiante (10 – 30°C) entre deux et cinq jours avant la manipulation au laboratoire de Biologie Moléculaire et d’Immunologie (BIOLIM) de la Faculté des Sciences de Santé de l’Université de Lomé.

- Prélèvement veineux

Un prélèvement sanguin sera effectué sur deux tubes contenant de l’éthylène diamine tétra-acétique (EDTA) pour le comptage des lymphocytes TCD4 et la mesure de la charge virale VIH. Ces prélèvements étaient acheminés à 4°C dans un délai de 4 heures au laboratoire BIOLIM. Le comptage des lymphocytes TCD4 sera effectué immédiatement, puis un plasma sera recueilli après centrifugation sur le second tube, aliquoté et conservé à – 80°C, pour la mesure de la charge virale VIH.

- 1. **Analyses de laboratoire**
     1. **Recherche du VPH**

Le dépistage de l’infection à VPH sera réalisé après amplification par Polymerisation Chain Reaction (PCR) et hybridation des molécules d’acide désoxyribonucléique (ADN) de VPH. Le kit, Mix PCR et Phire ® Hot Start II ADN polymérase (MAD-003930MU-P-E-30, Master Diagnostica), a été utilisé pour l’amplification. Le produit de PCR obtenu a été ensuite hybridé en utilisant le kit VPH Direct Flow CHIP optimisé (MAD-003930M-H, Master Diagnostica), et en suivant le protocole fourni par le fabricant sur l’automate (e-BRID System ®). Ce système permet le dépistage et le génotypage de 36 types de HPV dont 18 à haut risque (16, 18, 26, 31, 33, 35, 39, 45, 51, 52, 53, 56, 58, 59, 66, 68, 73 et 82) et 18 à bas risque oncogène (6, 11, 40, 42, 43, 44, 54, 55, 61, 62, 67, 69, 70, 71, 72, 81, 84 et CP6108) [16].

- - 1. **Détection de la charge virale VIH**

La détection de la charge virale sera réalisée par des tests de RT-PCR quantitative Abbott Real-Time HIV-1 VL (Abbott molecular, IL, USA), et un système d’extraction manuel couplé à un système d’amplification et de détection m2000rt. La limite de détection était < 40 copies/mL [17,18].

Les femmes vivant avec le VIH et ayant une charge virale en VIH supérieure ou égale à 10 000 copies / mm^3^ après un an de traitement ont été décrites comme ayant un contrôle virologique insuffisant.

- - 1. **Comptage du taux de lymphocytes TCD4**

Le comptage des lymphocytes TCD4 (CD4) sera réalisé par un cytomètre de flux, Facscalibur (BD, Sciences, Franklin Lakes, NJ États-Unis 07417) chez les toutes les patientes.

**3.7 Variable à étudier**

- Données sociodémographiques : âge, sexe, état matrimonial, parité, gestité, activités génératrices de revenus, niveau d'éducation
- Epidémiologie moléculaire du VPH
- Diversité génétique chez les co-infectés VIH-VPH

**3.8 Traitement des données**

**3.8.1 Saisie des données**

Les données seront saisies dans une base Excel.

**3.8.2 Analyse des données**

Les données seront saisies dans une base de données élaborée sous Microsoft Excel et analysées avec le logiciel STATA® version 14.1 (StataCorp, College Station, Texas, USA). Les résultats seront présentés sous forme d’effectifs et de proportions pour les variables qualitatives et de moyennes ou médianes pour les variables quantitatives. La prévalence de l’infection à VPH, par exemple, sera présentée avec son intervalle de confiance à 95%. La comparaison des variables qualitatives sera effectuée avec le test de Chi2 ou le test exact de Fischer et la comparaison des variables quantitatives sera faite avec le test t de Student ou le test d’analyse de variance ou les tests non paramétriques de Kruskal Wallis ou Wilcoxon. Le seuil de significativité sera fixé à 5%.

**3.9 Aspects éthiques**

Ce protocole sera soumis au Comité Ethique afin d’obtenir son avis favorable pour sa réalisation.

Une autorisation sera demandée aux directeurs des centres de soins médicaux pour les PVVIH avant sa réalisation. Seuls les patients ayant donné leur consentement et signé une fiche de consentement éclairé seront recrutés.

1. **Chronogramme**

La collecte et le traitement des données et des échantillons se fera entre Septembre 2014 et Décembre 2015 au TOGO.

1. **Archivage**

Les données seront archivées à BIOLIM-FSS/UL dans le compte du PNLS et du Ministère de la Santé.

**Références bibliographiques**

1. Sun XW, Ellerbrock TV, Lungu O, Chiasson MA, Bush TJ, Wright TC. Human papillomavirus infection in human immunodeficiency virus-seropositive women. Obstet Gynecol. mai 1995;85(5 Pt 1):680‑6.

2. Walboomers JM, Jacobs MV, Manos MM, Bosch FX, Kummer JA, Shah KV, et al. Human papillomavirus is a necessary cause of invasive cervical cancer worldwide. J Pathol. sept 1999;189(1):12‑9.

3. Ferlay J, Soerjomataram I, Dikshit R, Eser S, Mathers C, Rebelo M, et al. Cancer incidence and mortality worldwide: sources, methods and major patterns in GLOBOCAN 2012. Int J Cancer. 1 mars 2015;136(5):E359-386.

4. Pisani P, Parkin D, Bray F, Ferlay J. Erratum: Estimates of the worldwide mortality from 25 cancers in 1990. Int. J. Cancer, 83, 18-29 (1999). Int J Cancer. 10 déc 1999;83(6):870‑3.

5. Schaffer P, Sancho-Garnier H, Fender M, Dellenbach P, Carbillet JP, Monnet E, et al. Cervical cancer screening in France. Eur J Cancer. nov 2000;36(17):2215‑20.

6. Rousseau A, Bohet P, Merlière J, Treppoz H, Heules-Bernin B, Ancelle-Park R. Evaluation du dépistage organisé et du dépistage individuel du cancer du col de l’utérus : utilité des données de l’Assurance maladie. BEH. 7 mai 2002;(n°19):4.

7. Agence nationale pour le développement de l’évaluation médicale. Pratique des frottis cervicaux pour le dépistage du cancer du col. In: Recommandations et références médicales. Tome 2. Paris: Andem; 1995. p. 9–24. In.

8. Monsonego J. Dépistage du cancer du col utérin. Récents progrès et perspectives. In: Traité des infections et pathologies génitales à papillomavirus [Internet]. Springer, Paris; 2007 [cité 25 mai 2018]. p. 119‑36. Disponible sur: https://link.springer.com/chapter/10.1007/978-2-287-72066-6_14

9. Weekly epidemiological record. No. 15, 2009, 84, 117–132 http://www.who.int/wer [Internet]. [cité 7 janv 2019]. Disponible sur: https://www.who.int/wer/2009/wer8415.pdf

10. UNAIDS_Global_Report_2013_fr_1.pdf [Internet]. [cité 7 janv 2019]. Disponible sur: http://www.unaids.org/sites/default/files/media_asset/UNAIDS_Global_Report_2013_fr_1.pdf

11. WHO_HIV_2013.9_fre.pdf [Internet]. [cité 7 janv 2019]. Disponible sur: http://apps.who.int/iris/bitstream/handle/10665/85328/WHO_HIV_2013.9_fre.pdf;jsessionid=528A9E135EA5FE6E7CE7A65DB585AFBC?sequence=1

12. Smith JS, Melendy A, Rana RK, Pimenta JM. Age-specific prevalence of infection with human papillomavirus in females: a global review. J Adolesc Health. oct 2008;43(4 Suppl):S5-25, S25.e1-41.

13. Ault KA, Future II Study Group. Effect of prophylactic human papillomavirus L1 virus-like-particle vaccine on risk of cervical intraepithelial neoplasia grade 2, grade 3, and adenocarcinoma in situ: a combined analysis of four randomised clinical trials. Lancet. 2 juin 2007;369(9576):1861‑8.

14. WHO. Human Papillomavirus Vaccines: WHO Position Paper. WEEKLY EPIDEMIOLOGICAL RECORD. 2009;84(15):117‑32.

15. Djigma FW, Ouédraogo C, Karou DS, Sagna T, Bisseye C, Zeba M, et al. Prevalence and genotype characterization of human papillomaviruses among HIV-seropositive in Ouagadougou, Burkina Faso. Acta Trop. mars 2011;117(3):202‑6.

16. Herraez-Hernandez E, Alvarez-Perez M, Navarro-Bustos G, Esquivias J, Alonso S, Aneiros-Fernandez J, et al. HPV Direct Flow CHIP: a new human papillomavirus genotyping method based on direct PCR from crude-cell extracts. J Virol Methods. oct 2013;193(1):9‑17.

17. Tang N, Huang S, Salituro J, et al. A RealTime HIV-1 viral load assay for automated quantitation of HIV-1 RNA in genetically diverse group M subtypes A-H, group O and group N samples. *J Virol Methods* 2007; 146: 236–245.

18. Wang S, Xu F, Demirci U. Advances in developing HIV-1 viral load assays for resource-limited settings. *Biotechnol Adv* 2010; 28: 770–781.

**Annexes**

**Notice d’information**

**Contexte**

L’association entre le cancer du col et le Virus du Papillome Humain (VPH) est bien connue puisque le VPH a été reconnu comme étant impliqué dans 99,7% des cas de cancers du col. De nombreuses études ont mis en évidence une prévalence élevée de la coinfection à VPH avec l’infection à VIH, avec une augmentation à la fois des infections à VPH latentes et des infections symptomatiques.

**Ce qu’il faut savoir à propos des infections à Papillomavirus Humain et à VIH :**

- Le cancer du col utérin est l’un des rares cancers humains évitables. En effet, sa prévention repose sur le diagnostic très précoce des lésions bénignes ou précancéreuses dont le traitement rend en principe impossible le développement d’un cancer.
- Le cancer du col est la deuxième cause de cancers féminins dans le monde et représente environ 10 % de la totalité des cancers. Le nombre de cancers invasifs était estimé à 493 000, 83 % de ces cancers étant observés dans les pays en voie de développement. C’est la première cause de décès par cancer chez la femme dans les pays en voie de développement et la 10ème cause de décès dans les pays développés.
- L’Afrique sub-saharienne, reste aussi la région la plus touchée par l’infection à VIH depuis sa découverte il y a trente ans. L’épidémie du SIDA demeure toujours un problème de santé publique.

Les infections génitales à VPH comme l’infection à VIH sont principalement transmises par contact sexuel, le plus souvent mais pas exclusivement lors d’un rapport avec pénétration. Les VPH sont hautement transmissibles et la plupart des hommes et des femmes sexuellement actifs contracteront une infection à VPH, à un moment ou à un autre de leur vie. Tandis que la plupart des infections à VPH sont transitoires et bénignes, une infection génitale persistante par certains génotypes viraux peut conduire au développement de lésions précancéreuses et de cancers ano-génitaux. Il existe plus de 100 génotypes VPH. Certains sont associés à une immortalisation et à une transformation des cellules liées à la cancérogenèse. Parmi eux, au moins 13 sont susceptibles de provoquer un cancer du col ou sont associés à d’autres cancers ano-génitaux et oropharyngiens.

Vous devez faire un dépistage systématique de ses infections et informer votre médecin de tous les risques éventuels que vous aviez pris. Votre médecin est votre premier conseiller.

**Questions posées par notre étude**

On voudrait savoir si les génotypes qui circulent dans notre pays, sont identiques dans la population générale et chez les personnes infectées par le VIH puis si ces génotypes sont à haut risque oncogène ou à faible risque et enfin chez les jeunes filles avant leur vaccination voir si elles auront la protection de la souche vaccinale contre les génotypes qui circulent dans le pays.

**Déroulement de l’étude**

Si vous acceptez de participer à cette étude, il n’y aura pas de frais supplémentaire pour vous, sur votre prise en charge habituelle. La seule différence sera qu’aujourd’hui on vous prendra un tube de sang de 5 ml de plus que d’habitude, et vous ferez un prélèvement cervico-vaginal. Sur ce tube de sang, on dépistera le VIH et/ou on mesurera la charge virale, puis on fera un test de résistance si-possible. Enfin, sur le prélèvement cervico-vaginal, on recherchera le Papillomavirus Humain et le génotype. Les résultats de ces examens pourront servir à votre meilleure prise en charge. En plus, les résultats de cette étude serviront à la communauté toute entière, parce qu’ils feront progresser les connaissances sur la meilleure manière de suivre médicalement les personnes qui sont infectées par l’un ou les deux virus dans notre pays.

Tout ce qui est écrit ici vous sera re-expliqué par le médecin ou par l’assistant social : n’hésitez pas à poser toutes les questions que vous voudrez pour avoir plus de précisions sur les points que vous ne trouvez pas clairs. Si après ces explications vous avez envie de participer à cette étude, nous vous demanderons de bien vouloir signer le formulaire de consentement, qui indique que vous avez bien compris tout ce qui est écrit ici.

# Formulaire de consentement

**NOM :**

**Prénom :**

**IDENTIFIANT UNIQUE TG VPH 2015 :**

Le médecin désigné ci-dessous m’a proposé de participer à l’étude « **prévalence et diversité génétique du Virus du Papillome Humain (VPH) et coïnfection VPH – VIH au Togo**», selon ce qui est décrit dans la note d’information.

J’ai lu cette note et je l’ai comprise.

J’en ai discuté avec ce médecin qui m’a expliqué les avantages et les inconvénients de cette étude.

J’ai notamment bien compris que je suis libre d’accepter ou de refuser cette proposition, sans en être inquiété(e) et en continuant à bénéficier des mêmes conditions de traitement et de suivi.

J’accepte donc librement de participer à cette étude.

J’autorise que les données confidentielles qui me concernent soient consultées et analysées par les personnes qui collaborent à la recherche et qui sont tenues au secret médical.

Fait à .................

Le:/....../......./…...

Signature

Je soussigné, Dr  , certifie avoir expliqué à la personne susnommée l’intérêt et les modalités de participation à notre étude. Je m’engage à faire respecter les termes de ce formulaire de consentement, les droits et libertés individuels ainsi que les exigences d’un travail scientifique.

Fait à :...........

Le:/....../......./…..

Etude TG VPH 2015 Centre de santé : ……… le…../…../………../ IDENFICATION DU PATIENT N° du patient :………………………….. Code du centre de santé : N° patient pour étude TG VPH 2015 :……

**DONNEES GENERALES SUR LE PATIENT**  1. Date de naissance :…../…../…….../ 2. DDR:……..

3. Gestité :….. 4. Parité : ……

5. Nombre d’enfants vivants :….. 6. Nombre d’enfants décédés : ….

**CARACTERISTIQUES SOCIO-DEMOGRAPHIQUES**  7. Niveau d’instruction du patient : Primaire Secondaire Supérieur Autre 8. Statut matrimoniale actuel du patient : Célibataire Union libre Marié Séparé veuf Divorcé Autre Préciser…………………….

**STUATION SOCIO-ECONOMIQUE**  9. Le patient a-t-il des revenus : Oui / Non Si Oui : Revenus ponctuels ou Revenus réguliers 10.Type d’activité du patient : Sans emploi Contrat privé Fonctionnaire Etudient Retraité

**TRAITEMENT**

A demander au patient : 11. Avez-vous bénéficié déjà d’ARVs : O/N Préciser…………………

12. Avez-vous bénéficié précédemment de la PTME ? O/N Préciser…………………

13. Prenez-vous présentement des ARVs ? O/N Préciser…………………

**DONNEES BIOLOGIQUES**

14. Dosage dernier CD4 : Oui/ Non Préciser…………………

**Diagnostic VIH**

15. Type de VIH : VIH-1, VIH-2, Non déterminé

16. Mesure charge virale : Oui/ Non Préciser…………………

17. Test génotypique de résistance : Oui/ Non Préciser…………………

**Diagnostic VPH**

18. Présence VPH : Oui/ Non/ Non déterminé

19. Génotype VPH : Oui/ Non Préciser…………………

20. Type VPH : Haut risque / Faible risque Préciser…………………

**Couverture vaccinale anti-VPH**

21. Cervarix (anti-VPH 16, 18) : Oui/ Non Préciser…………………

22. Gardasil anti-VPH 6, 11, 16, 18) : Oui/ Non Préciser…………………

23. Gardasil 9 (anti-VPH 6, 11, 16, 18, 31, 33, 45, 52, 58) : Oui/Non Préciser…………………
